# Meta-Analysis of Preventive and Therapeutic Effects of Ligustrazine on Airway Remodeling in Asthmatic Rats

**DOI:** 10.1155/2022/3076377

**Published:** 2022-05-27

**Authors:** Yu-shuang Hua, Wen-qian Shang, Xia Chen, Ai-min Zhang

**Affiliations:** Department of Traditional Chinese Medicine, Zibo Maternal and Child Health Hospital, Zibo, Shandong 255000, China

## Abstract

**Objective:**

To assess the effect of ligustrazine on airway remodeling in asthmatic rats.

**Methods:**

To collect studies on the effects of ligustrazine on airway remodeling in asthmatic rat models, PubMed, Embase, CBM, Cochrane, Chinese Knowledge Infrastructure (CNKI), VIP, and Wanfang data (WANFANG) were searched using a computer. Two investigators independently screened the literature, extracted the data, and assessed the methodological quality by complying with the inclusion criteria. Moreover, a meta-analysis was conducted by adopting Stata 11.0.

**Results:**

On the whole, 10 articles were included. As indicated from the meta-analysis, we have the following: ① ligustrazine was capable of reducing the thickness of the airway smooth muscle and inhibiting the proliferation of smooth muscle (WMD = −5.98, 95% CI (−7.75, −4.42), *P* ≤ 0.001); ② ligustrazine could reduce the thickness of the airway wall and mitigate tracheal stenosis (WMD = 0.12, 95% CI (0.05, 0.20), *P* ≤ 0.001); ③ ligustrazine could decrease the number of eosinophils in the lung tissue and reduce airway inflammation (WMD = −14.47, 95% CI (−18.09, −10.86), *P* ≤ 0.001).

**Conclusion:**

Ligustrazine was demonstrated to be an effective therapeutic drug in asthmatic rats by preventing and treating airway remodeling. Further high-quality experimental studies should be conducted to investigate the mechanism of ligustrazine action in depth.

## 1. Introduction

Asthma refers to a chronic inflammation of the airways that is characterized by the infiltration of inflammatory cells (e.g., eosinophils, mast cells, and T lymphocytes) into the airways [1]. Its major pathogenesis involves airway inflammation, airway hyper-reactivity, and airway remodeling. Over the past few years, the incidence of asthma has shown a year-by-year growth. Though the GINA protocol for asthma has been issued each year, the improvement of clinical symptoms remains unsatisfactory. To be specific, the proportion of adults suffering from severe wheezing has increased from 1% to 21%, while that of children aged between 6 and 7 years is 20% [2].

The pathophysiological process in asthma ultimately causes airway remodeling to be altered. Airway remodeling is defined as recurrent airway injury and repair, thereby leading to structural variations in the airway wall. It is recognized as the critical pathological variation in diseases (e.g., bronchial asthma, chronic obstructive pulmonary disease, and pulmonary fibrosis) [3]. Its vital manifestations cover the proliferation of the airway smooth muscle and airway wall thickening, which can lead to airway stenosis, pulmonary function to deteriorate increasingly, and airway obstruction to aggravate gradually, thereby resulting in a significantly reduced quality of life of patients. As reported in recent studies, airway inflammation and remodeling occur simultaneously [4]; i.e., airway remodeling is present at the early stage of asthma. In addition, according to relevant studies, asthma patients with poor clinical prognosis are generally accompanied by severe airway remodeling [5]. Thus, inhibition of airway remodeling is of more significance to controlling asthma. Existing studies confirm the presence of eosinophils in the bronchial mucosa and pulmonary lumen during asthma, which has become a primary characteristic of asthma. And considerable activated eosinophils were found to accumulate in the induced sputum and bronchoalveolar lavage fluid of asthmatic patients [6]. As suggested by Wu et al. [7], eosinophils in induced sputum were higher in patients suffering from severe asthma than in patients with mild to moderate diseases, and they were higher in patients with acute attacks than in patients in remission, which demonstrated that the number of eosinophils could be consistent with the severity of asthma. Accordingly, the thickness of the airway smooth muscle, the thickness of the tracheal wall, and the number of eosinophils in the lung tissue were used to assess the effect exerted by ligustrazine on airway remodeling.

Glucocorticoids have been extensively applied for treating asthma and exert a certain effect on airway inflammation and airway remodeling, whereas the long-term use of hormones has certain side effects. By conducting a meta-analysis of 19 studies, Zhao et al. [8] found that inhaled glucocorticoids had the side effect of growth inhibition during the treatment of mild to moderate childhood asthma. Moreover, steroid-insensitive refractory asthma turns out to be a difficult problem for clinicians. Traditional Chinese medicine (TCM) treatment is a unique way to prevent and treat asthma in China, and numerous TCMs have achieved good results in the treatment of asthma. However, the pharmacological mechanism of these TCMs is unclear, and some studies have proposed that ligustrazine can inhibit the secretion of related inflammatory factors, mitigate airway inflammation, and improve airway hyperreactivity in asthmatic patients [9]. Ligustrazine is now considered a novel drug for treating asthma [10].

Most animal models of asthma are currently being explored, and rats, mice, and guinea pigs are being used to sensitize and model with ovalbumin (OVA). For this reason, inflammatory cell exudation, significant eosinophil infiltration, alveolar wall thickening, alveolar telangiectasia, and blood stasis will occur in the lung tissue after modeling, which complies with the characteristics of inflammatory responses in asthma [11]. On that basis, the authors of this study retrieved randomized controlled trials (RCTs) on the effects of ligustrazine on airway remodeling in OVA-sensitized rat models for the systematic assessment of its efficacy with quantitative indicators, as an attempt to lay an animal research basis for subsequent clinical trials.

## 2. Materials and Methods

### 2.1. Search Strategy

To search for studies on the effects of ligustrazine on airway remodeling in asthmatic rat models, PubMed, Embase, CBM, Cochrane, Chinese Knowledge Infrastructure (CNKI), VIP, and Wanfang data (WANFANG) were searched by computer from the database establishment to May 2021. The retrieval was achieved by combining subject headings with free words. English search terms were as follows: “Asthmas,” “Bronchial Asthma,” “Asthma, Bronchial,” “Ligustrazine,” “chuanxiongzine,” “TMPZ,” “tetramethyl pyrazine,” “tetramethylpyrazine hydrochloride,” “Liqustrazine.” Chinese search terms were as follows: “Xiao chuan,” “Chuanxiongzine,” “2, 3, 5, 6-tetramethylpyrazine,” “tetramethylpyrazine,” “chuanxiong.”

### 2.2. Inclusion and Exclusion Criteria

#### 2.2.1. Inclusion Criteria

The inclusion criteria were as follows: ① the article should be a RCT; ② the study subjects included Wistar rats or SD rats or BALB/c mice or guinea pigs with successful asthma modeling; ③ the intervention measures were intraperitoneal injection of ligustrazine in the asthma model as the experimental group and intraperitoneal injection of normal saline or no additional treatment in the asthma model as control; and ④ studies had complete data, or data could be obtained through other ways.

#### 2.2.2. Exclusion Criteria

The exclusion criteria were as follows: ① the outcome measures did not include the thickness of the airway smooth muscle, the thickness of the tracheal wall, and the number of eosinophils in the lung tissue; ② no control had outcome measures; ③ experiments included non-Wistar rats or SD rats or BALB/c mice or guinea pigs; ④ the intervention measure was the combination of ligustrazine and other substances; ⑤ clinical studies and review literature; ⑥ there was repeated published literature, and at least one study was retained by complying with the literature information and included in the meta-analysis; ⑦ the experimental data could not be extracted from the text, or the experimental results failed to obtain their specific data due to the use of graphical representation, and the original authors could not provide comprehensive data; ⑧ there were conference papers and abstracts.

### 2.3. Outcome Measures

The outcome measures are as follows: ① the thickness of the airway smooth muscle; ② the thickness of the tracheal wall; ③ the eosinophil number in the lung tissue.

### 2.4. Literature Extraction

Two investigators independently screened the literature by complying with the inclusion and exclusion criteria and extracted and collated the baseline data of the included studies. The following information was included: the title of the literature, first author, publication time, rat breed, modeling method, intervention grouping, number of cases in each group, study type, intervention time, and outcome measures. After the literature extraction was completed, two investigators cross-checked the results. For disagreement arising during the process, a third investigator would assist in the judgment.

### 2.5. Statistical Analysis

The meta-analysis was conducted by adopting Stata 11.0, and standardized mean differences with 95% confidence intervals (CIs) were exploited for continuous variables. The heterogeneity among the included article results was assessed by *I*^2^. When *I*^2^ < 50%, the heterogeneity among all articles was proved relatively low and the fixed-effect model was applied; when *I*^2^ > 50%, the heterogeneity among all studies would be proved to be high and the random-effect model was used. Subgroup analysis was conducted to explore the source of heterogeneity. Publication bias was judged according to the funnel plot and Egger's test results.

## 3. Results

### 3.1. Literature Search Results

**Figure 1:**
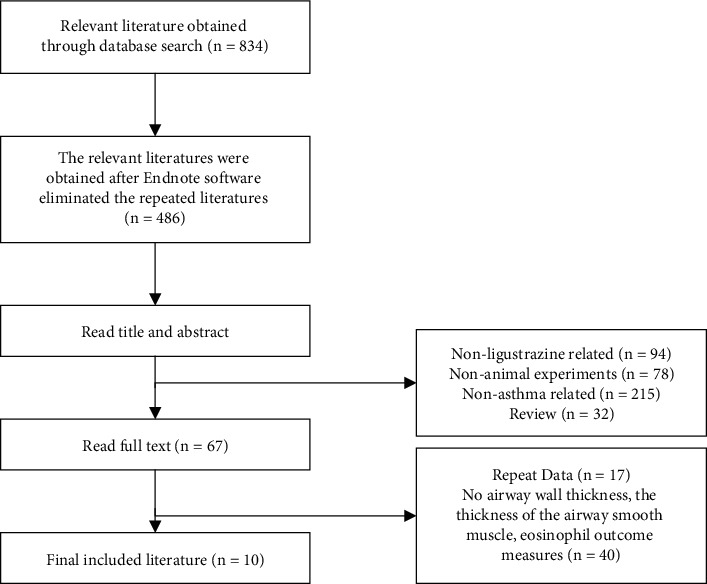
Flow chart of selection.

Based on the search strategy, 834 relevant articles were retrieved, 348 repeated articles were excluded, 94 nonligustrazine-related and ligustrazine combined with other drug treatments were excluded, 78 nonanimal experiments were excluded, 215 nonasthma articles were excluded, and 32 review articles were excluded. The other 67 articles were excluded for the repeated data publication (17 articles) and those without the thickness of the airway wall, the thickness of the airway smooth muscle, and eosinophil outcome measures (40 articles) by reading the full text. Lastly, 10 articles were included.  Figure 1 illustrates the specific literature screening.

### 3.2. Risk Assessment

**Figure 2:**
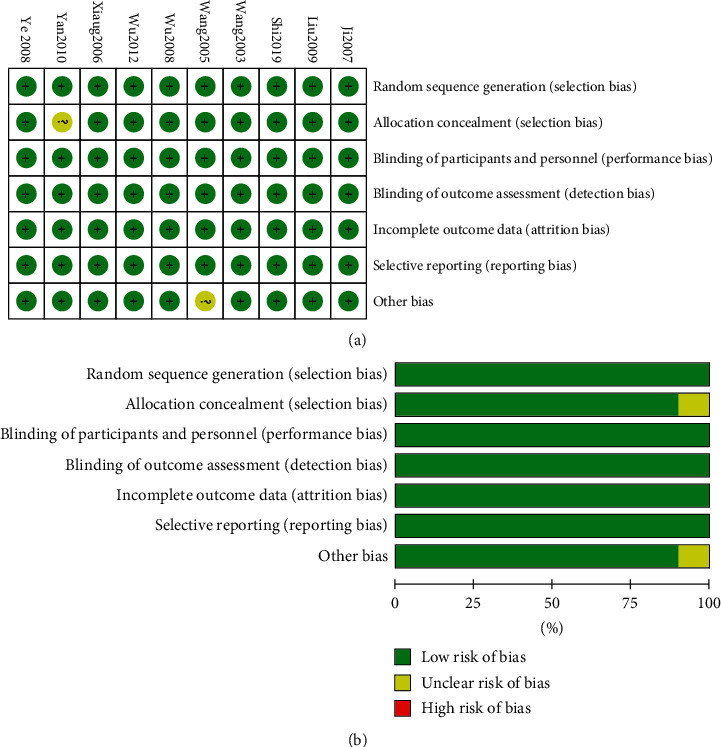
Risk bias evaluation of included studies.

In accordance with the Cochrane Collaboration's risk of bias assessment criteria [12], the quality of all articles included was assessed. If all items in the assessment criteria were at low risk of bias, the literature quality would be recorded as Level A; if one or more items were at medium risk of bias, it would be recorded as Level B; and if one or more items were high risk of bias, it would be recorded as Level C, as illustrated in Figure 2.

### 3.3. Basic Characteristics of the Literature

A total of 10 RCTs [13–22] were included in our meta-analysis, which involved 44 animals in the experimental group and 42 animals in the control group. To be specific, four articles used SD rats, four articles used Wistar rats, and two articles adopted BALB/c mice. OVA sensitization was used as a modeling method in all studies, and the course of observation reached 6–42 days. The outcome measures included the thickness of the airway smooth muscle, the thickness of the tracheal wall, and the number of eosinophils in the lung tissue. Given the Cochrane Collaboration's criteria for risk of bias assessment, the basic characteristics and quality of the literature of all included articles were assessed (Table 1). There are 8 articles rated as A and 2 articles rated as B.

### 3.4. Meta-Analysis Results

#### 3.4.1. Meta-Analysis Results of the Thickness of the Airway Smooth Muscle

**Figure 3:**
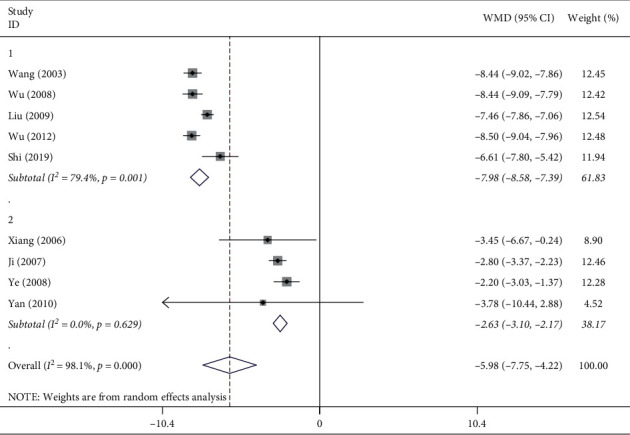
Meta-analysis of the effect of ligustrazine on airway smooth muscle thickness in asthmatic mice.

A total of 9 [13, 15–22] articles reported the effect exerted by ligustrazine on the thickness of the airway smooth muscle (Figure 3). As shown in Figure 3, ligustrazine significantly inhibited the thickening of the airway smooth muscle (WMD = −5.98, 95% CI (−7.75, −4.42), *P* ≤ 0.001) with high heterogeneity (*I*^2^ = 98.1%). As suggested from the literature reading, the high heterogeneity might be attributed to the different statistical methods for the determination of thickness of the airway smooth muscle in the literature, of which five articles [13, 17, 19, 21, 22] used direct measurement of 3–4 values and took the average. Four articles [15, 16, 18, 20] adopted the basement membrane perimeter for standardization, and the ratio of (smooth muscle outer edge area-smooth muscle inner edge area)/bronchial basement membrane perimeter was used to express the thickness of airway smooth muscle. The heterogeneity was significantly reduced in two subgroups: the direct measurement group (*I*^2^ = 79.4%) and the ratio-standardized group (*I*^2^ = 0.0%). The direct measurement of the group shows thicker airway smooth muscle in mice (WMD = 7.98, 95% CI (8.58, 7.39), *P* < 0.01), while the ratio-standardized group shows finer airway smooth muscle in mice (WMD = −2.63, 95% CI (−3.10, −2.17), *P*=0.629). This suggests that different measurement methods may be the cause of high heterogeneity. The heterogeneity of the direct measurement group was lower than before, but still >50%, which might be related to the different types of mice selected, the dose of ligustrazine injection, and the course of medication.

#### 3.4.2. Meta-Analysis Result of the Thickness of the Tracheal Wall

**Figure 4:**
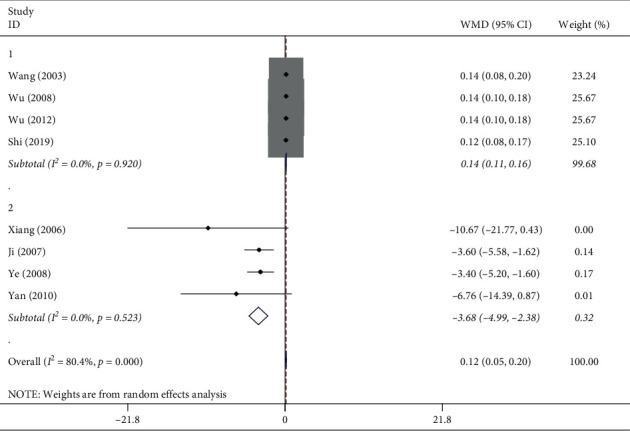
Meta-analysis of the effect of ligustrazine on airway wall thickness in asthmatic mice.

A total of 8 articles [13, 15–18, 20–22] reported the effect exerted by ligustrazine on airway wall thickness (Figure 4). As shown in Figure 4, with all data, *I*^2^ = 80.4% (WMD = 0.12, 95% CI (0.05, 0.20), *P* ≤ 0.001), which revealed high heterogeneity but statistical significance; i.e., ligustrazine could inhibit airway wall thickening. As revealed from the literature reading, the high heterogeneity might be due to different statistical methods, of which four articles [13,17,21,22] adopted the inner diameter/outer diameter of the airway wall and the larger the ratio, the thinner the airway wall. The other four articles [15, 16, 18, 20] adopted the wall area/endobronchial perimeter; the smaller the ratio, the thinner the airway wall thickness would be. According to the different statistical methods, it was divided into two subgroups: the inner/outer diameter ratio group and the area/perimeter ratio group. We ran the analysis again. The results showed the following: in the inside/outside diameter ratio group, *I*^2^ = 0.0% (WMD = 0.14, 95% CI (0.11, 0.16), *P*=0.920); in the area/perimeter ratio group, *I*^2^ = 0.0% (WMD = −3.68, 95% CI (−4.99, −2.38) *P*=0.523). The results showed that heterogeneity decreased significantly after grouping, and different statistical methods might be the reason for the increase in heterogeneity.

#### 3.4.3. Meta-Analysis of the Eosinophil Count in the Lung Tissue

**Figure 5:**
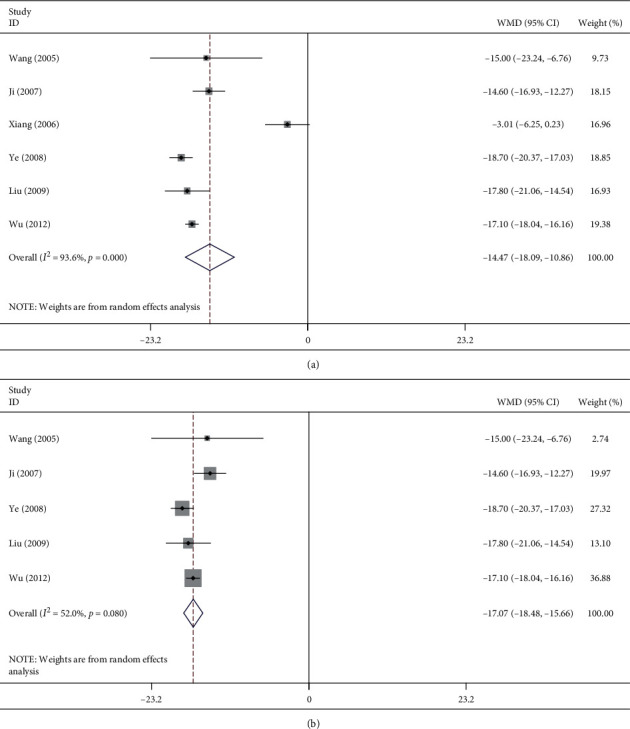
Meta-analysis of effect of ligustrazine on eosinophilic cells in lung tissues of asthmatic mice.

On the whole, six articles [14–16, 18, 19, 21] reported the effect exerted by ligustrazine on eosinophils in the lung tissue (Figure 5). As shown in Figure 5(a), with all data, *I*^2^ = 93.6%, which revealed high heterogeneity but statistical significance; i.e., ligustrazine could inhibit the proliferation of eosinophils in the lung tissue (WMD = −14.47, 95% CI (−18.09, −10.86), *P* ≤ 0.001). The high heterogeneity might be attributed to the fact that one literature [15] used the proportion of eosinophils in bronchoalveolar lavage fluid to express the proliferation of eosinophils and the other articles adopted the eosinophil count of lung tissue. Then, as shown in Figure 5(b), after excluding the article using the proportion of eosinophils [15], the heterogeneity of the analysis was significantly reduced (*I*^2^ = 52.0%). And, the inhibitory effect of ligustrazine on the proliferation of eosinophils in the lung tissue was more obvious (WMD = −17.07, 95% CI (−18.48, −15.66), *P* ≤ 0.001).

### 3.5. Analysis of Literature Publication Bias

**Figure 6:**
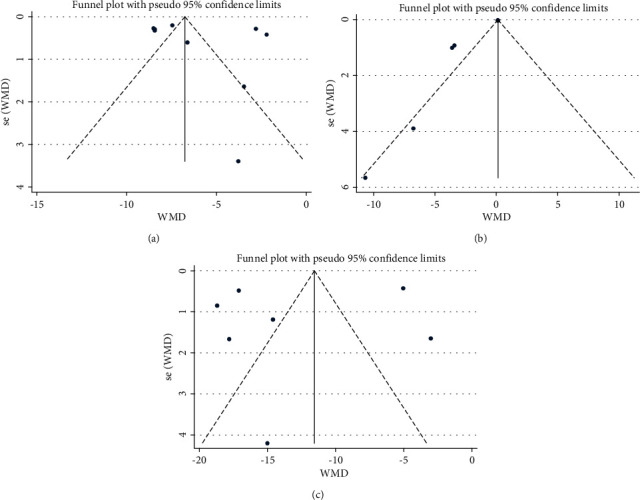
Funnel plot for data analysis of three indicators. (a) Funnel plot of airway smooth muscle thickness. (b) Funnel plot of gas tube wall thickness. (c) Funnel plot of eosinophils in the lung tissue.

As shown in Figure 6, the funnel plots of three analysis indicators seem not symmetrical, indicating that there may be public bias. Therefore, as shown in Table 2, we further did Egger's test and found that the *P* values of the thickness of airway smooth muscle and the number of eosinophils were both greater than 0.1, indicating that there was no publication bias. However, the data of air duct wall thickness (*P*=0.01) suggested publication bias, which might be related to the fact that the ratio of inner and outer diameter of the middle airway wall was too close in the literature.

## 4. Discussion

TCM has thousands of years of clinical application history in China and exerts unique therapeutic effects on preventing and treating asthma with few side effects. TCM believes that prolonged illness leads to stasis. Recurrent asthma has the pathological basis of lung collateral stasis. Therefore, the treatment of asthma should promote blood circulation to remove blood stasis, collaterals, and asthma. *Ligusticum chuanxiong hort*, with its warm nature and strong fragrance, is capable of “improving microcirculation, activating blood stasis and dredging blood vessels,” which is recognized as a good blood-activating and stasis-dissipating drug. Its chemical composition primarily comprises phthalein derivatives, alkaloids, and phenolic acid compounds, of which the amide alkaloid tetramethylpyrazine pharmacological effects and clinical application research are more abundant [23]. Ligustrazine (chemical name: 2, 3, 5, 6-tetramethylpyrazine), an amide alkaloid isolated and extracted from Chuanxiong, refers to a novel calcium antagonist with various effects (e.g., dilating blood vessels, inhibiting platelet aggregation, preventing thrombosis, and improving cerebral ischemic symptoms [24]), which has been extensively used for cardiovascular and cerebrovascular, renal, and pulmonary multisystem diseases. Yang et al. [25] conducted an analysis by using the text mining method. They reported Kechuanning, ligustrazine injection, and western medicine dexamethasone as the most commonly used Chinese patent medicines and western medicine for treating asthma with a combination of traditional Chinese and western medicines, respectively, which could improve microcirculation, activate blood stasis, and dredge blood vessels.

It has been suggested that ligustrazine could inhibit the synthesis of collagen and reduce the thickening of the reticular basement membrane layer while inhibit the thickening of the smooth muscle layer, thereby hindering the thickening of the airway wall [26]. Wang et al. [27] found that ligustrazine could significantly improve carbamylcholine-induced airway hyper-reactivity and reduce IgE levels in the serum of asthmatic rats, which demonstrated that ligustrazine may have a positive effect in preventing and treating acute attacks of asthma. According to the research by Zhang et al. [28], ligustrazine exerted a significant relaxant effect on the resting tension of airway smooth muscle and histamine-induced hypertension in guinea pigs. Cheng et al. [29] reported that ligustrazine combined with ipratropium bromide could significantly increase clinical efficacy, improve pulmonary function, and reduce inflammatory responses. However, the specific mechanism of action of ligustrazine remains unclear, and Shi et al. [30] suggested that ligustrazine might facilitate airway remodeling in asthmatic mice by regulating the TGF-*β*/Smad signaling pathway. Furthermore, Chang et al. [31] concluded that ligustrazine could mitigate airway inflammation and oxidative stress in asthmatic mice and that the mechanism might be associated with AMPK/NF-*κ*B and Nrf-2/HO-1 signaling pathways.

The pathophysiology of airway remodeling is that chronic airway inflammatory stimulation leads to airway smooth muscle proliferation and airway wall thickening, resulting in airway stenosis and airflow restriction. Therefore, three indicators (i.e., the thickness of the airway smooth muscle, the thickness of the tracheal wall, and the number of eosinophils in lung tissue) were selected as evaluation standards in this study. We conducted a meta-analysis of the 10 included articles. As indicated from the results, the overall heterogeneity of the respective index was high, whereas P was less than 0.01, which demonstrated that ligustrazine could effectively inhibit the proliferation of the airway smooth muscle, reduce airway inflammation, and decrease the number of eosinophils in lung tissue, thereby inhibiting airway remodeling. Data analysis showed that the heterogeneity of each index was high. Further subgroup analysis supported that different measurement methods may be the main reason for the high heterogeneity. However, the data of airway smooth muscle thickness was of high heterogeneity, and there was a publication bias in the data of airway wall thickness. We consider the following possible reasons: ① Different species of mice were selected, and there were racial differences. ② Although the researchers used OVA for sensitization, the time, route, and dose of sensitization were inconsistent, which may cause different degrees of airway inflammation. ③ There are limited samples in the literature. ④ Injections of ligustrazine were inconsistent. ⑤ Unequal duration of medication. ⑥ The treatment methods of the control group were inconsistent. Standardized and unified experimental procedures should be developed in the future, and the number of samples needs to be expanded to lower heterogeneity and publication bias.

The limitations of this study are elucidated as follows. ① The included articles are the basic study of animal experiments. However, there is still a lack of standardized methodological studies on the meta-analysis of animal experiments. Thus, it is urgent to establish and constantly improve the assessment system of basic experimental studies of animals and enhance their comparability with clinical studies. ② Among the included articles, publication bias exists in the airway wall thickness index, which may be related to the fact that the study sample size is excessively small and the ratio of inner and outer diameters of the airway wall is extremely close; a large sample and high-quality literature study should still be included in the analysis to achieve more reliable study results. ③ The guinea pig is the most suitable animal model to prepare the allergic type. However, there is a lack of indicators of the thickness of the airway smooth muscle and the thickness of the tracheal wall in guinea pigs in the current studies. Further relevant models should be prepared for the subsequent observations. ④ There is a lack of molecular data, such as the effects of ligustrazine on transforming growth factor-*β* and GATA-3. At present, there are few relevant data, which cannot be systematically evaluated. Further observations can be made in subsequent experiments.

In brief, ligustrazine exerts a significant effect on preventing and treating airway remodeling in asthma, which is instructive for further clinical studies. However, its mechanism of action remains unclear, and there are few relevant data for statistical analysis. Future experimental studies should be conducted to investigate the mechanism of ligustrazine in depth. Due to the limited quality and number of included articles and certain publication bias of some results, the conclusion of this study requires to be verified by more high-quality studies.

## Figures and Tables

**Table 1:** Basic features of included studies.

Included articles	Rat breed	Number of subjects (test/control)	Method of excitation	Interventions		Treatment course	Observation indicators	Literature level
				Test	Control			
Wang [13]	SD rat	10/10	Aerosol inhalation of 1% OVA solution was performed for 20 min, once daily for the first 3 consecutive days and then every other day	Intraperitoneal injection of ligustrazine 80 mg/kg 30 min before excitation	Equal volume of normal saline	4 weeks	1, 2	A

Wang [14]	SD rat	8/8	Aerosol inhalation of 1% OVA solution was performed for 20 min, once daily for the first 3 consecutive days and then every other day	Intraperitoneal injection of ligustrazine 80 mg/kg 30 min before excitation	Equal volume of normal saline	4 weeks	3	B

Xiang [15]	BALB/c mouse	10/10	Aerosol inhalation of 1% OVA solution for 30 min, once daily	Intraperitoneal injection of ligustrazine 80 mg/kg 1 h before excitation	Untreated	7 days	1, 2, 3	A

Ji [16]	SD rat	12/12	Aerosol inhalation of 1% OVA solution for 30 min, once daily	Intraperitoneal injection of ligustrazine 80 mg/kg 30 min before excitation	Equal volume of normal saline	2 weeks	1, 2, 3	A

Wu [17]	Wistar rat	6/6	Aerosol inhalation of 1% OVA solution for 30 min, once daily	Intraperitoneal injection of ligustrazine 5 mg/kg 30 min before excitation	Untreated	4 weeks	1, 2	B

Ye [18]	SD rat	10/10	Aerosol inhalation of 1% OVA solution for 20 min, once daily	Intraperitoneal injection of ligustrazine 60 mg/kg 30 min before excitation	Untreated	7 days	1, 2, 3	A

Liu [19]	Wistar rat	8/6	Aerosol inhalation of 1% OVA solution for 30 min, once daily	Intraperitoneal injection of ligustrazine 80 mg/kg 30 min before excitation	Untreated	4 weeks	1, 3	A

Yan [20]	Wistar rat	10/10	10 mg OVA and 20 mg aluminium hydroxide was prepared into 2 ml mixed atomization inhalation for 10 min, once daily	Intraperitoneal injection of ligustrazine 2 mg 1 h before excitation	Untreated	8 weeks	1, 2	A

Wu [21]	Wistar rat	6/6	Aerosol inhalation of 1% OVA solution for 30 min, once daily	Intraperitoneal injection of ligustrazine 5 mg 30 min before excitation	Untreated	4 weeks	1, 2, 3	A

Shi [22]	BALB/c mouse	10/10	2 mg/ml OVA solution was instilled into the nose	Intraperitoneal injection of ligustrazine 80 mg/kg 30 min before excitation	Untreated	6 days	1, 2	A

Note: (1) the thickness of the airway smooth muscle; (2) the thickness of the tracheal wall; (3) the eosinophil number in the lung tissue.

**Table 2:** Egger's test results of three groups of data.

Analysis indicators	Egger's test *P* values
Airway smooth muscle thickness	0.542
The thickness of the tracheal wall	0.01
Eosinophils in the lung tissue	0.736

## Data Availability

All the data generated and analyzed during this study are included within this article. The datasets supporting the conclusion in this study are available in a public database from PubMed, Embase, CBM, Cochrane, Chinese Knowledge Infrastructure (CNKI), VIP, and Wanfang data (WANFANG).
